# Association Tests of Multiple Phenotypes: ATeMP

**DOI:** 10.1371/journal.pone.0140348

**Published:** 2015-10-19

**Authors:** Xiaobo Guo, Yixi Li, Xiaohu Ding, Mingguang He, Xueqin Wang, Heping Zhang

**Affiliations:** 1 Department of Statistical Science, School of Mathematics & Computational Science, Sun Yat-Sen University, Guangzhou, GD 510275, China; 2 SYSU-CMU Shunde International Joint Research Institute, Shunde, GD 528300, China; 3 Department of Biostatistics, Yale University School of Public Health, New Haven, CT 06520, United States of America; 4 Southern China Research Center of Statistical Science, Sun Yat-Sen University, Guangzhou, GD 510275, China; 5 Zhongshan School of Medicine, Sun Yat-Sen University, Guangzhou, GD 510080, China; 6 State Key Laboratory of Ophthalmology, Zhongshan Ophthalmic Center, Sun Yat-Sen University, Guangzhou, GD 510080, China; 7 Peking University HSBC Business School, Shenzhen, GD 518055, China; Indiana University Bloomington, UNITED STATES

## Abstract

Joint analysis of multiple phenotypes has gained growing attention in genome-wide association studies (GWASs), especially for the analysis of multiple intermediate phenotypes which measure the same underlying complex human disorder. One of the multivariate methods, MultiPhen (O’ Reilly et al. 2012), employs the proportional odds model to regress a genotype on multiple phenotypes, hence ignoring the phenotypic distributions. Despite the flexibilities of MultiPhen, the properties and performance of MultiPhen are not well understood, especially when the phenotypic distributions are non-normal. In fact, it is well known in the statistical literature that the estimation is attenuated when the explanatory variables contain measurement errors. In this study, we first established an equivalence relationship between MultiPhen and the generalized Kendall tau association test, shedding light on why MultiPhen can perform well for joint association analysis of multiple phenotypes. Through the equivalence, we show that MultiPhen may lose power when the phenotypes are non-normal. To maintain the power, we propose two solutions (ATeMP-rn and ATeMP-or) to improve MultiPhen, and demonstrate their effectiveness through extensive simulation studies and a real case study from the Guangzhou Twin Eye Study.

## Introduction

Genome-wide association studies (GWASs) have emerged as a common tool for identifying the genetic variants for numerous complex diseases. The conventional GWASs focus on a single phenotype, aiming to identify the associations between single nucleotide polymorphisms (SNPs) and a univariate phenotype [1–3]. However, complex human disorders, such as mental disorders, are often characterized by multiple intermediate phenotypes [4, 5]. In addition, many phenotypes, such as body-mass-index and refractive error, are derived from other measurements [6, 7]. Modeling the association between multiple phenotypes and a genetic variant may reveal a weak or moderate genetic association that is not apparent from single phenotype GWASs, increasing statistical power and providing fruitful biological insights by identifying pleiotropic variants [8–10].

In recent years we have witnessed an increasing interest in multiple phenotypes GWASs. Among the numerous multivariate methods that have been proposed, some commonly used ones include canonical correlation analysis (CCA) [11], MANOVA [12], and the linear mixed model [13, 14]. However, these methods are highly dependent on the normality assumption, and are known to inflate Type I error [15, 16] when the phenotypic distributions deviate from normality. To deal with this problem, MultiPhen employs the proportional odds model by modeling the genotype score as an ordinal response and the multiple phenotypes as predictors, aiming to identify a combination of phenotypes associated with the genotype. This method ignores the fact that the phenotypes are measured with uncertainty, and hence avoids the need to make a distributional assumption on the phenotypic distributions [16]. Nonetheless, extensive simulations suggest that MultiPhen is one of the most powerful multivariate methods [17].

Despite the promising performance of MultiPhen, the properties of MultiPhen are not well understood. One exception is a recent work by Wang [18] that offered an explicit expression of the score test statistic for MultiPhen and provided some insights into how MultiPhen works in the multiple phenotypes association analyses. Here, we prove that the score test in MultiPhen is in fact equivalent to the generalized Kendall’s tau association test [19], and hence is really an alternative presentation of a method established earlier. Thus, it is not surprising that MultiPhen works well for the multivariate analysis under certain circumstances. Using the equivalence formula to the generalized Kendall’s tau statistic, we demonstrate that MultiPhen may have poor power when the phenotypes are non-normal. To maintain robust power, we propose two solutions to improve MultiPhen or the generalized Kendall’s tau when the phenotypes are non-normal.

The rest of this paper is organized as follows. First, we establish the equivalence between MultiPhen and the generalized Kendall’s tau association test, and demonstrate that the MutiPhen may lose power for non-normal phenotypes. Second, we propose two association tests for multiple phenotypes (ATeMP) that perform well even when the phenotypes are non-normal. Finally, extensive simulations and real GWAS data are used to evaluate the performance of ATeMP.

## 1 Materials and Methods

### 1.1 Notation

Suppose that there are *n* subjects in an association study. Let (**Y**_*i*_, *G*_*i*_) denote the observed data of the *i* ^*th*^ subject, where **Y**_*i*_ = (*Y*_*i*1_, …, *Y*_*iK*_)^*T*^ is a vector of *K* phenotypes of the *i* ^*th*^ individual and *G*_*i*_ is the genotypic score. For simplicity, we consider a single variant and the genotypic score is coded as 0, 1, or 2, corresponding to the number of minor alleles in a biallelic locus.

### 1.2 MultiPhen

MultiPhen uses the proportional odds logistic regression to model the probability distribution of an individual’s genotype *G*_*i*_ as a function of the multiple phenotypes,

$$P\left(G_{i}\leq g\right)=\frac{1}{1+\exp(-\alpha_{0}-\sum_{k=1}^{K}\alpha_{k}Y_{ik})},$$ (1)

where the *α*’s are regression coefficients. Under this setting, the score test statistic is [18]

$$S_{1}=\frac{1}{n\left(1-\bar{\pi_{0}}\right)\left(1-\bar{\pi_{1}}\right)\left(1-\bar{\pi_{2}}\right)}W^{T}V^{-1}W,$$ (2)

where

$$W=\left(1-\bar{\pi_{0}}\right)\sum_{i:g_{i}=0}Y_{i}+\left(\bar{\pi_{2}}-\bar{\pi_{0}}\right)\sum_{i:g_{i}=1}Y_{i}+\left(\bar{\pi_{2}}-1\right)\sum_{i:g_{i}=2}Y_{i},$$ (3)

$$V=n^{-1}\sum_{i=1}^{n}\left(Y_{i}-\bar{Y}\right){\left(Y_{i}-\bar{Y}\right)}^{T},$$ (4)

and $\bar{\pi_{0}}$, $\bar{\pi_{1}}$ and $\bar{\pi_{2}}$ are the proportions of genotype *G* with values of 0, 1, and 2, respectively. The statistic *S* follows a chi-square distribution with degrees of freedom *df* = *K*.

### 1.3 The generalized Kendall’s tau and the equivalence

The generalized Kendall’s tau is one of the earliest association tests for multiple phenotypes [19]. Because it is a nonparametric test, it can be applied to a hybrid of continuous and ordinal phenotypes. Specifically, the generalized Kendall’s tau statistic can be defined as

$$U=\frac{2}{n(n-1)}\sum_{i>j}f_{g}\left(G_{i}-G_{j}\right)\;(\begin{array}{c} f_{1}\left(Y_{i1}-Y_{j1}\right)\\ \ldots \\ f_{K}\left(Y_{iK}-Y_{jK}\right) \end{array}),$$ (5)

where *f*_*g*_(⋅) and *f*_*k*_(⋅) are kernel functions. Two popular choices of the kernel function are the identity function and the sign function. For clarity, let *f*_*g*_ be the sign function because *G* is in an ordinal scale, and let *f*_*k*_(⋅) be the identity function. Then, statistic *U* can be simplified as

$$U=\frac{2}{n(n-1)}\sum_{i>j}sign\left(G_{i}-G_{j}\right)\left(Y_{i}-Y_{j}\right)\propto \sum_{i=1}^{n}{\bar{g}}_{i}Y_{i},$$ (6)

where

$${\bar{g}}_{i}=\frac{1}{n}\sum_{j=1}^{n}sign\left(G_{i},G_{j}\right)=\{\begin{array}{ccc} 1-\bar{\pi_{0}} & & \text{if}\;G_{i}=0,\\ \bar{\pi_{2}}-\bar{\pi_{0}} & & \text{if}\;G_{i}=1,\\ \bar{\pi_{2}}-1 & & \text{if}\;G_{i}=2. \end{array}$$ (7)

Conditional on the phenotypes, the generalized Kendall’s tau test statistic can be constructed as [19]

$$S_{2}=U^{T}\hat{\text{var}}\left(U|Y\right)U={\left(\sum_{i=1}^{n}{\bar{g}}_{i}Y_{i}\right)}^{T}(\hat{\text{var}}{\left(\sum_{i=1}^{n}{\bar{g}}_{i}Y_{i})\right)}^{-1}\left(\sum_{i=1}^{n}{\bar{g}}_{i}Y_{i}\right).$$ (8)

Note that $\sum_{i=1}^{n}{\bar{g}}_{i}Y_{i}=W$ defined in Eq (3), and as shown in the appendix,

$$\hat{\text{var}}\left(\sum_{i=1}^{n}{\bar{g}}_{i}Y_{i}\right)=\left(1-\bar{\pi_{0}}\right)\left(1-\bar{\pi_{1}}\right)\left(1-\bar{\pi_{2}}\right)\sum_{i=1}^{n}\left(Y_{i}-\bar{Y}\right){\left(Y_{i}-\bar{Y}\right)}^{T},$$ (9)

therefore the generalized Kendall’s tau test statistic *S*_2_ is equal to the score test statistic *S*_1_ of MultiPhen. Given the earlier work on the generalized Kendall’s tau, it is not surprising that MultiPhen works well for the multiple phenotypes association studies under various circumstances.

### 1.4 ATeMP

The MultiPhen used the classic technique in genetic analysis [20] by conditioning on the phenotypes, and avoided the need to assume phenotypic distributions. However, when the phenotypes are non-normal, MultiPhen may lose power. This is more convenient to see by examining the generalized Kendall’s tau. For example, when all phenotypes are continuous, the identity function is the most natural choice for the kernel function. It is known that this choice is not efficient in the absence of normality [21]. To maintain the power for testing the non-normally distributed phenotypes, we introduce two solutions for association tests of multiple phenotypes (ATeMP):

- ATeMP-rn: The idea is to replace the original phenotypes with their normalized ranks, a common approach to transforming non-normal data [14, 22]. Let (*R*_1*k*_, ⋯, *R*_*nk*_) be the rank vector of the *k* dimensional phenotypic vector (*Y*_1*k*_, …, *Y*_*nk*_). Next, we can employ the inverse normal transformation, and transform *Y*_*ik*_ into $Y_{ik}^{*}=\Phi^{-1}\left(\frac{R_{ik}}{n+1}\right)$. Then, we apply the MultiPhen or equivalently generalized Kendall’s tau.

When a phenotype is in an ordinal scale, the sign function is more suitable as the kernel function. And, if we assume the genetic effect is additive, the generalized Kendall’s tau statistic in Eq (6) can be simplified as

$$U\propto \sum_{i=1}^{n}G_{i}\;(\begin{array}{c} \frac{1}{n}\sum_{j=1}^{n}sign\left(Y_{i1}-Y_{j1}\right)\\ \ldots \\ \frac{1}{n}\sum_{j=1}^{n}sign\left(Y_{iK}-Y_{jK}\right) \end{array}),$$ (10)

which can be viewed as testing the association between *G*_*i*_ and and the transformed phenotypes:

$${\left(\frac{1}{n}\sum_{j=1}^{n}sign\left(Y_{i1}-Y_{j1}\right),\ldots ,\frac{1}{n}\sum_{j=1}^{n}sign\left(Y_{iK}-Y_{jK}\right)\right)}^{T}.$$ (11)

Note that $\frac{1}{n}\sum_{j=1}^{n}sign\left(Y_{ik}-Y_{jk}\right)$ can be regarded as the residual corresponding to *Y*_*ik*_ when the *k*th phenotype (*Y*_1*k*_, ⋯, *Y*_*nk*_) is ordinal [23]. Hence, we refer to this transformation as the “ordinal residual transformation,” which leads to the following improvement for MultiPhen:

- ATeMP-or: For a non-normally distributed phenotype, we employ the ordinal residual transformation as described above, and transform *Y*_*ik*_ into $Y_{ik}^{*}=\sum_{j=1}^{n}sign\left(Y_{ik}-Y_{jk}\right).$ Then, we apply the MultiPhen or equivalently generalized Kendall’s tau.

### 1.5 Simulation Study 1: Bivariate Phenotypes

We conducted simulation studies to systematically evaluate the efficiency as well as the robustness of ATeMP. We generated bivariate traits under the bivariate linear model

$$Y_{i1}=\beta_{G1}\;*\;G_{i}+\beta_{E1}\;*\;E_{i},$$ (12)

$$Y_{i2}=\beta_{G2}\;*\;G_{i}+\beta_{E2}\;*\;E_{i}+\epsilon ,$$ (13)

where *G*_*i*_ is the causal variant with minor allele frequency of 0.2, *E*_*i*_ is a random effect, and *ϵ* is the random error following *N*(0, *σ* ^2^). Varying the distribution of *E*_*i*_ among several non-normal distributions yields a variety of non-normal phenotypes. Specifically, we set *β*_*G*1_ = 0.1 and *β*_*G*2_ = 0, or 0.05, or 0.1, and considered the following different distributions for *E*_*i*_: (1)*N*(0, 1), (2)*t*(3), (3)*Laplace*(1.5, 1) and (4) *Gamma*(1, 2). We chose suitable values of *β*_*E*1_, *β*_*E*2_ and *σ* ^2^ such that the variances of both *Y*_*i*1_ and *Y*_*i*2_ are equal to 1 and the between-phenotype correlation, *r*, varies from -0.8 to 0.8 in an increment of 0.4.

To evaluate the statistical power, we simulated 1000 datasets under each simulation scenario above. Each simulated dataset consisted of 2000 unrelated individuals. The significance level was fixed at 5 × 10^−4^. This nominal level of significance is much higher than the typical level of significance in GWAS to reduce the computational time in simulation. However, we believe it is small enough for the purpose of comparing the power of MultiPhen, ATeMP-rn, and ATeMP-or.

We assessed the Type I error of these tests by letting MAF be 5%. 50000 datasets were simulated and the significance level was set to be 5 × 10^−4^ in this simulation study. To assess the asymptotic approximation, we also considered relatively small sample sizes of 300 and 500.

### 1.6 Simulation Study 2: High Dimensional Phenotypes

To further evaluate the efficiency and robustness of ATeMP, we considered high dimensional phenotypes. The phenotypes are generated using a linear additive model

$$Y_{k}=\beta_{k}G+\sqrt{a}U_{k}+\sqrt{1-a}\varepsilon_{k},k=1,\cdots ,K,$$ (14)

where (*U*_1_, ⋯, *U*_*K*_)^*T*^ follows multivariate normal distribution with mean 0 and covariance matrix Σ. A gradient of strong to low levels of correlation for Σ is simulated; that is, *ρ*_*ij*_ = 0.8^∣*i*−*j*∣^. Under the alternative hypothesis, we assumed that the genetic variant is associated with one third of the phenotypes. We simulated independent *ɛ*_*k*_ from one of the following distributions: (1) *N*(0, 1); (2)*t*(3); (3)*Laplace*(1.5, 1); (4)*Gamma*(1, 2). Finally, *a* was set to be 0.4 and the number of phenotypes *K* was set to be 5 and 10.

To evaluate the statistical power, we simulated 1000 datasets under each simulation scenario above. Each simulated dataset consisted of 1000 unrelated individuals. The significance level was fixed at 5 × 10^−4^. The minor allele frequency of the causal variant *G* is set to be 0.3. The genetic variant explains 0.3% of the phenotypic variations when *ɛ*_*k*_ follows the normal distribution, and 0.6% for the other distributions. We assessed the Type I error by simulating 50000 datasets, and the sample sizes were set to be 300, 500 and 1000.

### 1.7 Study of Myopia: Testing Candidate SNPs from Guangzhou Twin Project

**Fig 1:**
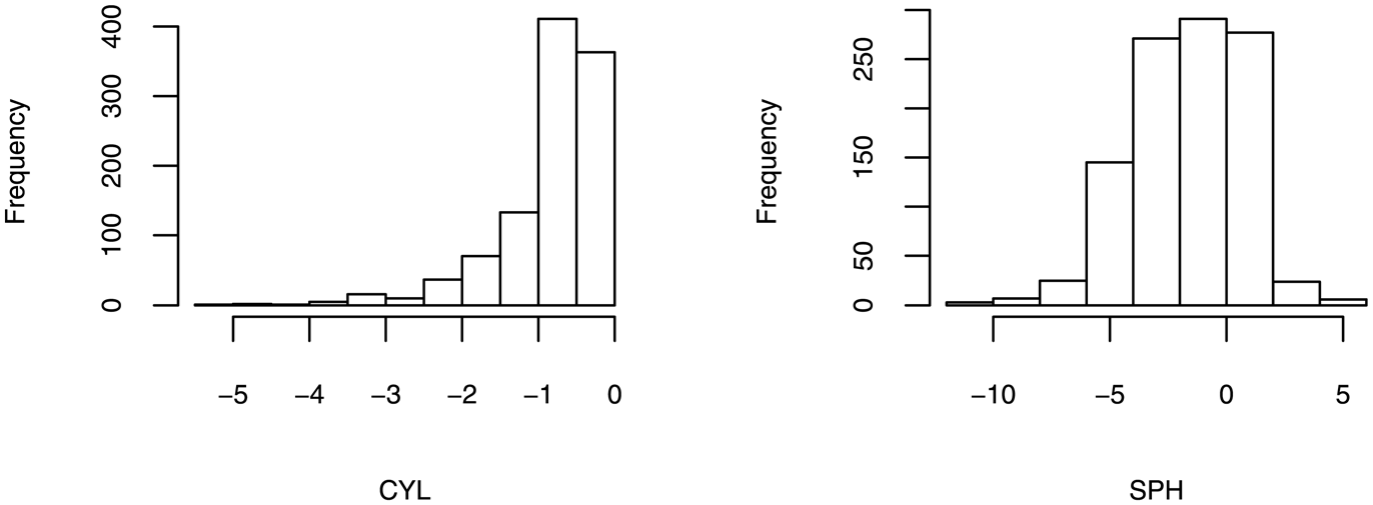
The Histograms of Phenotypes SPH and CYL.

Here, we applied MultiPhen, ATeMP-rn, and ATeMP-or to evaluated 38 candidate SNPs which are identified from three large GWASs [3, 24, 25] for refractive error. We analyzed a dataset from the Guangzhou Twin Eye Study, which iss a population-based registry designed to examine the genetic and environmental etiologies for myopia. It was launched in 2006, and has completed eight consecutive annual follow-up examinations, with more than 1200 twin pairs participating. In brief, twins born in Guangzhou aged 7 to 15 years received annual eye examinations from 2006 and on. The protocol and examination procedures have been published elsewhere [26]. Written, informed consent was obtained for all participants from either parents or guardians of the participating children after careful explanation of the study in detail, including the discussion and specific consent for the use of DNA information. Ethical committee approval was obtained from the Zhongshan University Ethical Review Board and Ethics Committee of Zhongshan Ophthalmic Center [26]. We focus on refractive error, which is the most common eye disorder in the world and is the leading cause of blindness [3]. Spherical lens (SPH) and cylindrical lens (CYL), two major intermediate traits of refractive error, have gained increasing interest in the GWAS [27]. Borrowing the strength of the multiple phenotypes association studies, in this report, we are interested in the the multiple phenotypes associations analysis for SPH and CYL. Fig 1 displays the distributions of SPH and CYL. We can observe that the distribution of CYL is heavily skewed, suggesting that transformed phenotypes would be preferrable before performing the association tests. Specifically, we employed both the inverse normal transformation and the ordinal residual transformation for CYL and SPH.

The current data are from the Guangzhou Twin Eye Study. A detailed description has been published elsewhere [26]. The GWAS data included 1055 individuals from the first-born twins. Age and gender were considered as covariates.

## 2 Results

### 2.1 Simulation Studies of Statistical Power and Type I Error

**Fig 2:**
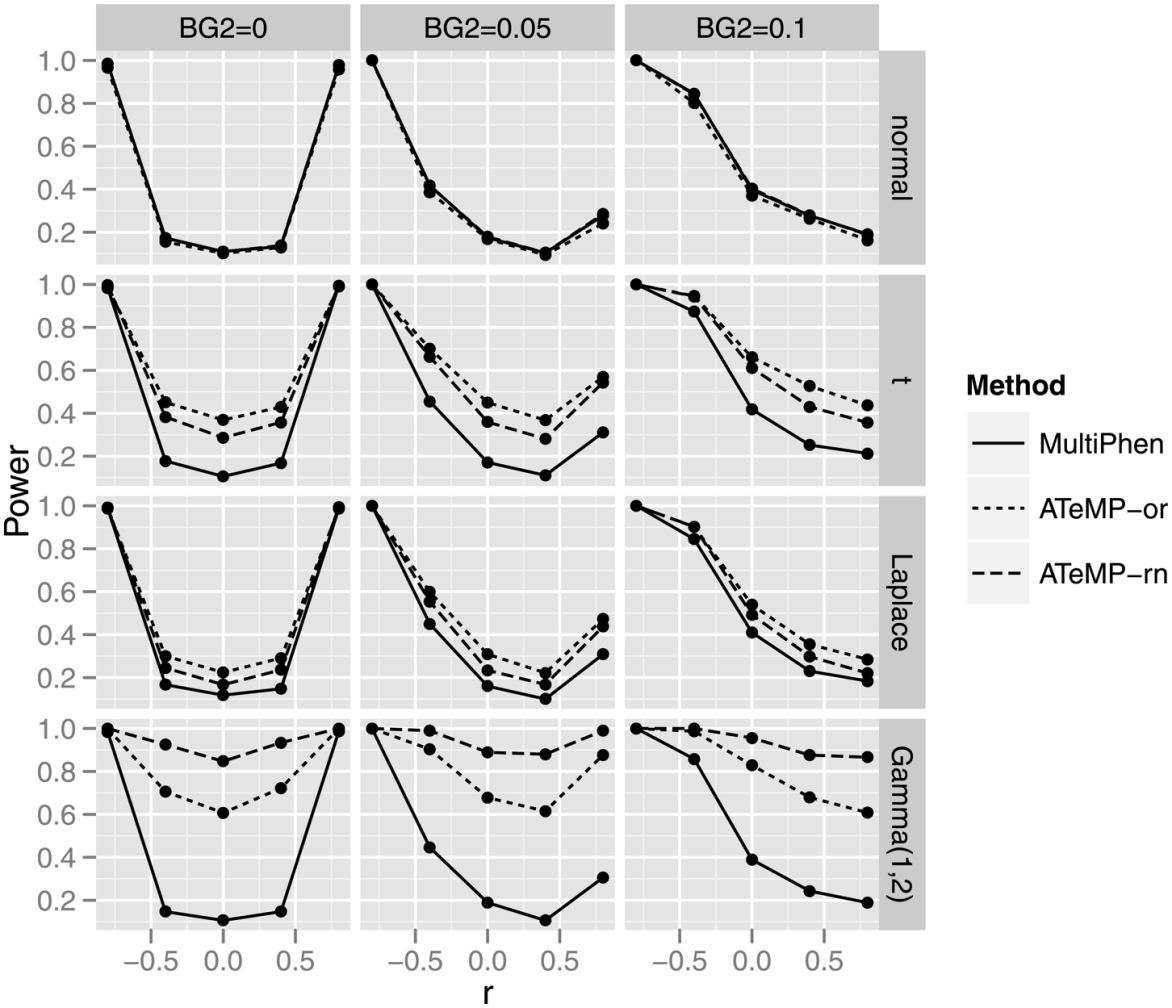
The power of the multiple phenotypes association tests at the significance level 5 × 10^−4^ under different simulation settings. Different type of lines represent different methods.

Fig 2 presents the power comparison under different simulation settings for bivariate phenotypes. We can learn from Fig 2 that MultiPhen can lose a great deal of power when the phenotypes are non-normal. The loss is more severe, as shown in Fig 2, when the phenotypes are heavily skewed such as from the Gamma distribution. However, ATeMP-rn and ATeMP-or can recover the loss. Table 1 displays the results of power comparisons under different simulation settings when the number of phenotypes are five and ten. Similarly to the power comparison for bivariate phenotypes, ATeMP-rn and ATeMP-or can recover the power loss when the phenotypes are non-normal. These simulations confirm that transforming non-normal phenotypes is necessary. Even though MultiPhen makes no assumption on the phenotypic distributions, it does not necessarily mean that it is efficient.

**Table 1:** The power of the multiple phenotypes association tests at the significance level 5 × 10^−4^ when the number of phenotypes are 5 and 10.

No.Phenotypes	Distribution	MultiPhen	ATeMP-nr	ATeMP-or
5	normal	0.63	0.63	0.55
	t	0.18	0.24	0.28
	Laplace	0.25	0.29	0.31
	Gamma(1,2)	0.23	0.36	0.37
10	normal	0.73	0.72	0.63
	t	0.56	0.75	0.80
	Laplace	0.41	0.45	0.48
	Gamma(1,2)	0.38	0.52	0.52

To offer a practical guide, we summarize the order of superiority between different methods. When the phenotypic distribution is heavily-tailed, such as the t distribution or the Laplace distribution, ATeMP-or is the most powerful approach in all of the considered simulation settings as can be seen clearly from Fig 2 and Table 1. When the phenotypic distribution is heavily skewed, such as the Gamma distributions, ATeMP-rn is the perferred method for the bivariate phenotypes. However, the performance of ATeMP-rn and ATeMP-or is almost the same when the phenotypes are high dimensional, such as five or ten in our simulation studies.

Table 2 reports the Type I error rates when the nominal significance level is set to be 5 × 10^−4^. We can observe that the Type 1 error rates of ATeMP-rn and ATeMP-or are very close to the nominal values, indicating that these methods can control Type I error well in the considered simulation settings. The Type 1 error rates of MultiPhen is inflated for the *t* distribution when the sample size is 300 or 500. We do not observe inflated Type 1 error rate for MultiPhen when the sample size is 2000. S1 Table also presents the Type 1 error rate when the number of phenotypes are 5 and 10. We can observe that all methods can control Type 1 error well in the considered simulation settings, indicating that the asymptotic distribution provides an adequate approximation for high dimensional phenotypes.

**Table 2:** Type I error of the multiple phenotypes association tests at the nominal significance levels of 5 × 10^−4^ when the between-phenotype correlation is 0.5 and the minor allele frequency of the tested locus is 5%. The sample sizes are set to be 300, 500 and 2000, respectively.

Sample Size	Distribution	MultiPhen	ATeMP-nr	ATeMP-or
300	normal	0.00052	0.00050	0.00034
	t	0.00082	0.00048	0.00056
	Laplace	0.00026	0.00036	0.00026
	Gamma(1,2)	0.00068	0.00054	0.00054
500	normal	0.00048	0.00052	0.00052
	t	0.00066	0.00046	0.00040
	Laplace	0.00038	0.00046	0.00044
	Gamma(1,2)	0.00062	0.00046	0.00042
2000	normal	0.00048	0.00042	0.00058
	t	0.00054	0.00054	0.00052
	Laplace	0.00056	0.00054	0.00048
	Gamma(1,2)	0.00042	0.00046	0.00038

### 2.2 Association Study on Myopia

In Table 3, we display the SNPs with p-value < 0.05 from the joint analysis. ATeMP-rn yields nearly the same results as ATeMP-or, and the most significant SNP (rs12229663 with p-value of 4.9 × 10^−4^) is identified by the ATeMP-or. For the SNPs with p-value < 0.01, most of the p-values from ATeMP are smaller than those from MultiPhen, suggesting again that transforming phenotypes is helpful in this real data analysis. These results confirm the observations from the simulation studies. For SNPs with p-value > 0.01 (Table 3 and S2 Table), there are no apparent benefits from ATeMP.

**Table 3:** P-values from association tests of jointly analyzing CYL and SPH. The bold-face texts highlight where ATeMP tests may be superior to MultiPhen.

SNP	MAF	Gene	MultiPhen	ATeMP-rn	ATeMP-or
rs12229663	0.45	PTPRR	2.1e-03	**6.4e-04**	**4.9e-04**
rs524952	0.42	GJD2	9.7e-03	**7.5e-03**	9.9e-03
rs7837791	0.48	TOX	1.8e-02	**5.0e-03**	**2.9e-03**
rs1881492	0.1	CHRNG	4.5e-02	2.3e-01	2.0e-01
rs1898585	0.36	PDE11A	4.7e-02	5.3e-02	7.9e-02

After the Bonferroni correction, no SNPs are significant by using MultiPhen. However, ATeMP-rn or ATeMP-or identified one significant SNP rs12229663.

## 3 Discussion

In this report, we first pointed out and prove that a recent method for multiple phenotypes association testing, MultiPhen, is in fact equivalent to an earlier test proposed for the same purpose. After establishing this equivalence, we demonstrated that MultiPhen suffers from a substantial loss of power when the phenotypic distributions were non-normal. This calls for the caution that the use of a distribution-free test may be convenient, but it may also be inefficient.

To recover the power loss of MultiPhen, we proposed two phenotypic transformations prior to the use of MultiPhen or the equivalent generalized Kendall’s tau. The first method, ATeMP-rn, employs the frequently used inverse normal transformation for the non-normal phenotypes before any association test. The second method, ATeMP-or, uses a particular form of residuals in a proportional odds model involving an ordinal response [23, 28]. Extensive simulations demonstrate that ATeMP tests can recover the power when the phenotypic distributions are heavy-tailed or highly-skewed, while MultiPhen suffers from a substantial loss of power. In addition, we also compared the power by using the permutation method rather than the asymptotic distribution. The results (S1 Fig) indicate again that transforming phenotypes is helpful when the phenotypic distributions are non-normal.

In our simulation studies, we observed that the power of the multivariate methods is high when the correlation of bivariate phenotypes is negative and the genetic effects on the individual phenotypes are positive. Others [13, 16, 29] have also noted this phenomenon that the power increases when the correlation of the phenotypes is in opposite direction to the phenotypic genetic effects. It can also be explained from the perspective of principle component analysis [29].

We applied MultiPhen and ATeMP tests to evaluate 38 candidate SNPs from the Guangzhou Twin Eye Study. Five SNPs showed nominally significant p-value (p-value<0.05), indicating that part of candidate SNPs of refractive error are associated with its two major intermediate traits. Our real data analysis confirmed that ATeMP tests are superior to MultiPhen, underscoring the usefulness of transforming the non-normal phenotypes prior to association testing, despite the fact that MultiPhen is distribution-free.

## Appendix: The derivation of $\hat{\text{var}}\left(\sum_{i=1}^{n}{\bar{g}}_{i}Y_{i}\right)$

We first note that

$$\sum_{i=1}^{n}{\bar{g}}_{i}=n\left(\left(1-\bar{\pi_{0}}\right)\bar{\pi_{0}}+\left(\bar{\pi_{2}}-\bar{\pi_{0}}\right)\left(1-\bar{\pi_{0}}-\bar{\pi_{2}}\right)\right)+\left(\bar{\pi_{2}}-1\right)\bar{\pi_{2}})=0$$

and

$$\begin{array}{cc} \hat{\text{var}}\left({\bar{g}}_{i}\right) & ={\left(1-\bar{\pi_{0}}\right)}^{2}\bar{\pi_{0}}+{\left(\bar{\pi_{2}}-\bar{\pi_{0}}\right)}^{2}\left(1-\bar{\pi_{0}}-\bar{\pi_{2}}\right)+{\left(\bar{\pi_{2}}-1\right)}^{2}\bar{\pi_{2}}\\ & =\left(1-\bar{\pi_{0}}\right)\left(1-\bar{\pi_{1}}\right)\left(1-\bar{\pi_{2}}\right) \end{array}$$

since $\bar{\pi_{0}}+\bar{\pi_{1}}+\bar{\pi_{2}}=1$. Therefore,

$$\begin{array}{cc} \hat{\text{var}}\left(\sum_{i=1}^{n}{\bar{g}}_{i}Y_{i}\right) & =\hat{\text{var}}\left(\sum_{i=1}^{n}{\bar{g}}_{i}\left(Y_{i}-\bar{Y}\right)\right)\\ & =\sum_{i=1}^{n}\left(Y_{i}-\bar{Y}\right)\hat{\text{var}}\left({\bar{g}}_{i}\right){\left(Y_{i}-\bar{Y}\right)}^{T}\\ & =\left(1-\bar{\pi_{0}}\right)\left(1-\bar{\pi_{1}}\right)\left(1-\bar{\pi_{2}}\right)\sum_{i=1}^{n}\left(Y_{i}-\bar{Y}\right){\left(Y_{i}-\bar{Y}\right)}^{T}. \end{array}$$

## Supporting Information

S1 Fig The power of the multiple phenotypes association tests at the significance level 5 × 10^−4^ under different simulation settings. Different types of curve represent different methods. The simulation settings are the same as the simulation studies for bivariate phenotypes in Section 1.5. To alleviate the computational burden, the sample size was set to be 500, and the significance level was set to be 0.05.
(EPS)

S1 Table Type I error of the multiple phenotypes association tests when the phenotypes are five and ten, respectively. The nominal significance level is set to be 5 × 10^−4^, and the sample sizes are set to be 300, 500 and 100, respectively.
(XLS)

S2 Table P-values from association tests of 38 candidate SNPs by jointly analyzing CYL and SPH. (XLS)
